# Supplementary figures and images for: Regionality of short and long period oscillators in the suprachiasmatic nucleus and their manner of synchronization
Source: PLoS One. 2022 Oct 18;17(10):e0276372. doi: 10.1371/journal.pone.0276372 (PMC9578605; doi:10.1371/journal.pone.0276372)

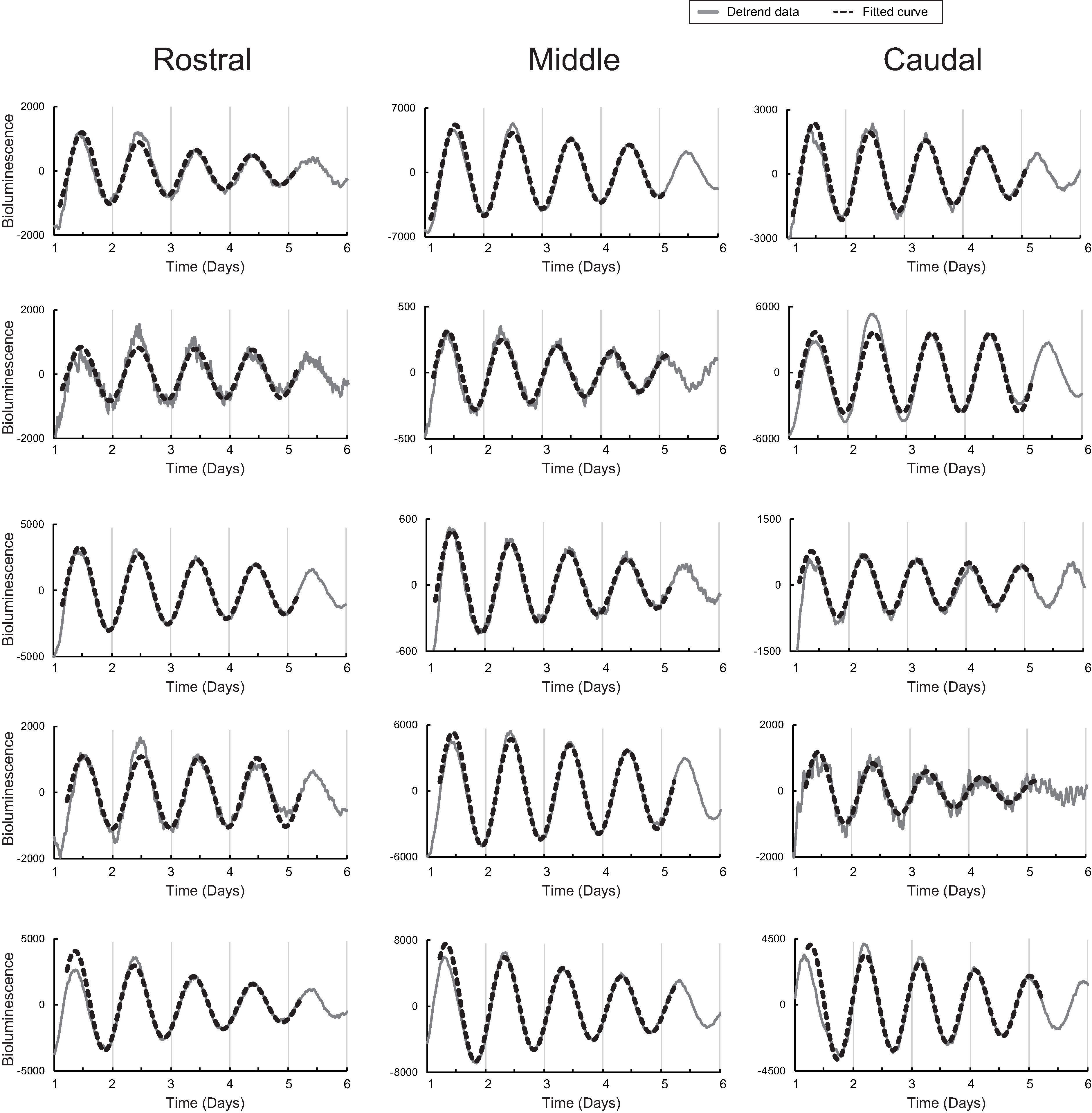

Supplement: S1 Fig — A representative of the six specimens is shown in Fig 2A. Data from five other SCNs are shown. The gray lines and black dotted lines indicate the detrended wave forms and fitted curves, respectively. (TIF) [file pone.0276372.s001.tif]

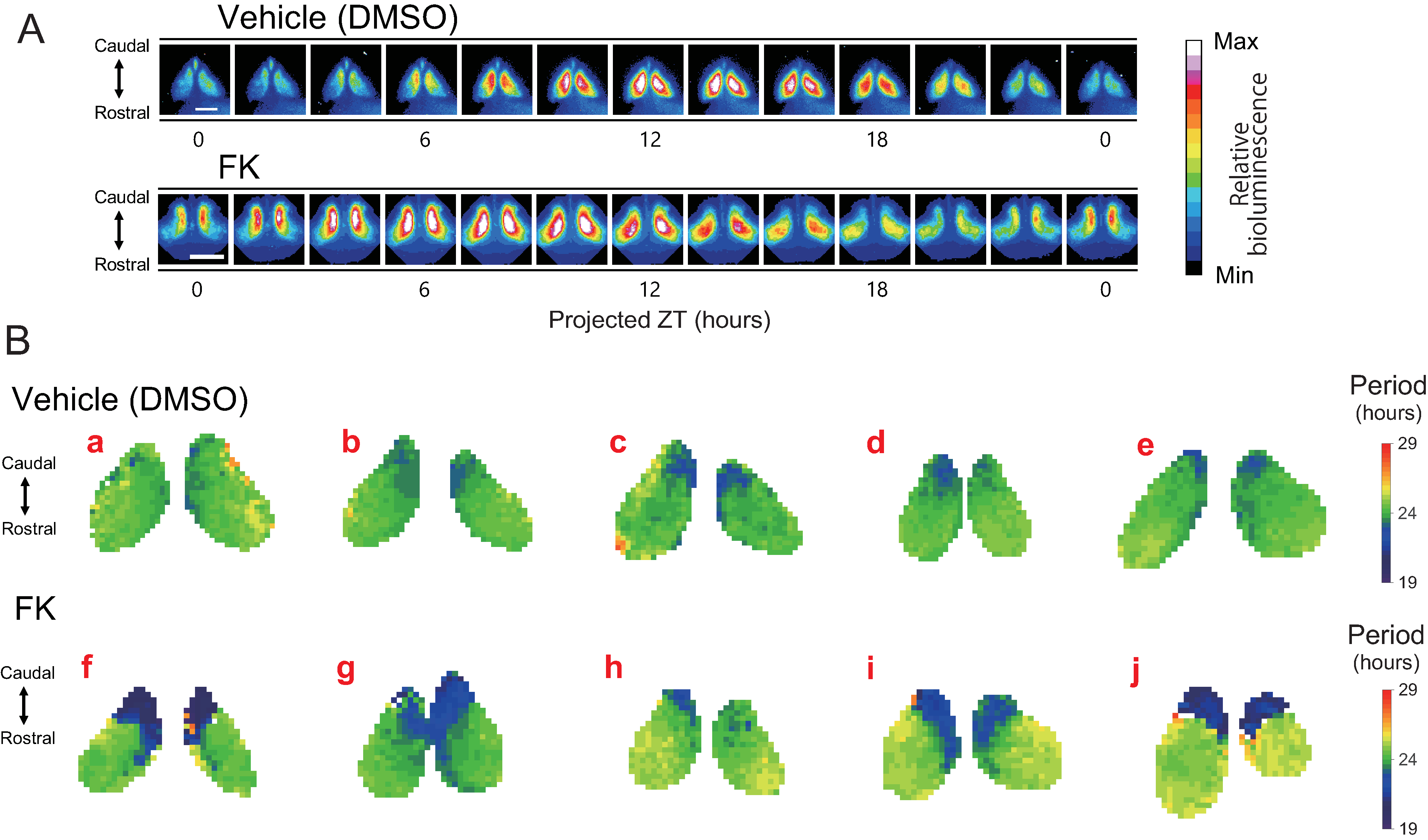

Supplement: S2 Fig — (A) Time series of bioluminescence images from SCN horizontal slices treated with vehicle (DMSO, upper panel) and forskolin (FK, lower panel). The numbers below the pictures indicate projected ZT (ZT, zeitgeber time). Scale bar: 500 μm. (B) Grid analysis of circadian periods of Per2::dLuc bioluminescence rhythms. Grid size: 32 μm. (TIF) [file pone.0276372.s002.tif]

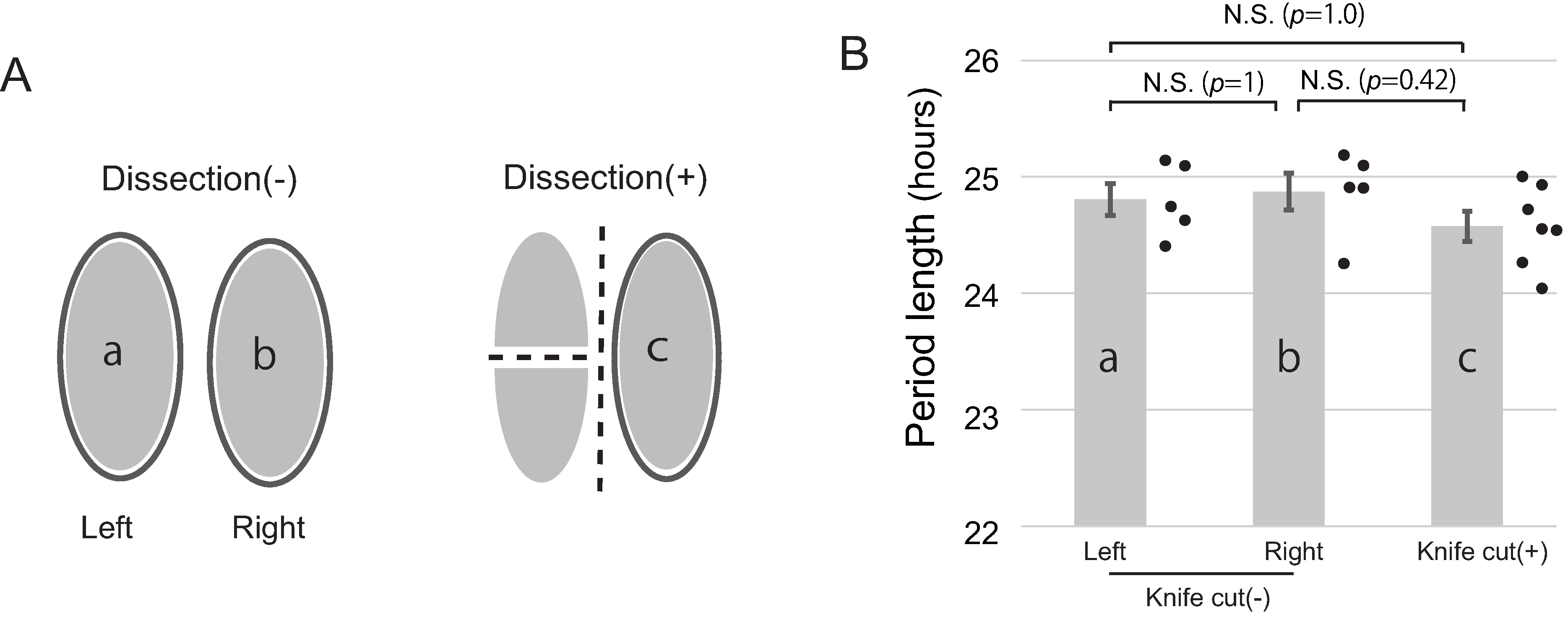

Supplement: S3 Fig — (A) ROIs were set on the SCN with and without dissection. Mean bioluminescence rhythm from each ROIs were measured. The periods of bioluminescence rhythms from the SCN without dissection (left (a) and right (b), left picture, Dissection(-), n = 5) and from unilateral SCN with dissection along the midline but without rostro-caudal dissection ((c), right picture, Dissection(+), n = 7). Dashed lines in the right picture indicate the dissection lines. (B) Statistical analysis between Dissection(-) and Dissection(+) groups. Bioluminescence period was 24.8 ± 0.14, 24.9 ± 0.16 hours for Dissection(-) group (Mean ± SE, Left and Right respectively), and 24.6 ± 0.13 hours for Dissection(+) group (Intact). No significant difference was found by repeated measures one-way ANOVA with a post hoc Bonferroni test between right and left SCN without dissection (a and b) and with dissection (c) (repeated measures one-way ANOVA; F(2,8) = 1.4, p = 0.31: post-hoc Bonferroni test; a vs. b, p = 1.0, a vs. c, p = 1.0, b vs. c, p = 0.42). (TIF) [file pone.0276372.s003.tif]
