# Supplementary material for: Regionality of short and long period oscillators in the suprachiasmatic nucleus and their manner of synchronization
Source: PLoS One. 2022 Oct 18;17(10):e0276372. doi: 10.1371/journal.pone.0276372 (PMC9578605; doi:10.1371/journal.pone.0276372)
Supplement: Attachment — Submitted filename: Response to Reviewers.docx [file pone.0276372.s007.docx]

Author‘s Response to Reviewers

First of all, we appreciate reviewers for their constructive and meaningful suggestions. According to reviewers’ suggestion, we revised Abstract and added Data availability statement. Point to point responses are shown as follows. Comments by editor and reviewers are attached with ## in front of the paragraph and responses by authors are without any signs.

**Reviewer #2**

\## *Relative to my response to Review Question 2: To allow for other possible interpretations of the data, I suggest that the authors qualify or moderate the statement in the Abstract "We found that the longer circadian rhythm of the rostral region entrains the circadian rhythm in the caudal region". A better statement might read "... the rostral region appears to entrain the circadian rhythm in the caudal region". I am concerned that a rigorous test of entrainment was not performed, which would require more sophisticated control of the interactions between the oscillators including a repeated control and release from entrainment and assessment of subsequent phase.*

Thank you for the valuable comment. We agree with your opinion and revised Abstract as follows: (Line 35-36) We found that the longer circadian rhythm of the rostral region appears to entrain the circadian rhythm in the caudal region.

\## *Also, I did not see a statement saying that all the data are available or would be made available in a data repository if the paper is accepted for publication.*

Thank you very much for your comment. We added Data availability statement in the manuscript.

Added as follows: (Line 438 - 442) Data used to generate the figures and supplementary figures in this manuscript are accessible on a public figshare repository (10.6084/m9.figshare.21261324). Other data and further information are also available from the corresponding author on reasonable request.

We sincerely appreciate your kind but critical readings and meaningful suggestions.
